# Development and Psychometric Evaluation of Bilingual English-Urdu Pakistani Critical Thinking Disposition Scale

**DOI:** 10.12669/pjms.39.5.6832

**Published:** 2023

**Authors:** Raisa Gul, Nuzhat Sultana, Gideon Victor, Ambreen Gowani, Huma Rubab

**Affiliations:** 1 Dr. Raisa Gul Professor and Dean, Shifa Tameer-e-Millat University, College of Nursing, Islamabad, Pakistan; 2 Dr. Nuzhat Sultana Former Director Nursing Education, Shifa International Hospital, Islamabad, Pakistan; 3 Gideon Victor Assistant Professor. Shifa Tameer-e-Millat University, College of Nursing, Islamabad, Pakistan; 4 Ambreen Gowani Senior Lecturer, Aga Khan University, Karachi - Pakistan; 5 Dr. Huma Rubab Assistant Professor, Shifa Tameer-e-Millat University, College of Nursing, Islamabad, Pakistan

**Keywords:** Critical thinking, Psychometric, Nurses, Nursing, Dispositions

## Abstract

**Objective:**

To develop and test the psychometric properties of the Pakistani Critical Thinking Dispositions Scale.

**Methods:**

In the item generation phase, constructs of the scale were identified through an in-depth literature review and items were written to measure the constructs. Following this, input of the experts was obtained for content validity index. In the item reduction phase, psychometric properties were measured. Initially, the scale was administered to 580 study participants during May 2018-2020 after approval from the institutional review board. Data was analyzed through SPSS v21, AMOS v21 and Omega extension.

**Results:**

First phase identified 54-items for seven constructs including contextual perspective, perseverance, reflection, intellectual integrity, creativity, open-mindedness, and inquisitiveness. Second phase determined KMO test value of 0.974 and Bartlett’s test (P-Value < 0.001). The second-order confirmatory factor analysis showed a good fit model explaining 73.37% total variance. Parsimony and baseline comparison indices were favorable. Alpha and Omega value of 42-items was 0.869.

**Conclusion:**

Pakistani Critical Thinking Dispositions scale owning seven constructs and 42-items is valid, reliable, and feasible to use in undergraduate nursing education. However, its utilization in other healthcare disciplines can be tested.

## INTRODUCTION

Critical thinking (CT) is a phenomenon of worldwide importance and a desired outcome in higher and professional education.^1^ Thus learning of CT skills is essential for students in 21^st^ century.^2^ Healthcare professionals require CT to learn and apply complex concepts in health care education and practice. Moreover, CT has accentuated as a every person’s need to survive in the rapidly changing world.^3^ Critical thinking is composite of skills and dispositions.^4^ Critical thinking dispositions (CTDs) refer to the habits of mind, virtues, or intellectual character that prompts people to exercise their critical thinking skills. Therefore, measuring CTDs is imperative for developing and exercising CT skills. The development of the Pakistani-Critical Thinking Disposition Scale (PCTDS) was grounded in four arguments. Firstly, CTDs are important for the quality of education and research.

Secondly, critical thinking scales and inventories are developed in contexts for validity of measurement. Therefore variations are evident in the constructs and number of items among CTD scales and inventories.^5^-^9^ The contextual knowledge is crucial for critical thinking and learning.^10^ Thirdly, due to lack of contextually developed scales and inventories, borrowing and adapting remain an option but it is not without financial and linguistic implications. Most established instruments often do not allow amendments. Furthermore, heavy remittance to use questionnaire is demanded. Fourthly, self-ratings of second language proficiency is a concern which could impact desired completion of questionnaire.^11^ This could produce pseudo effect and potential to haze the purity of research findings. That being the case, indigenously developed bilingual CTDs scale was undeniably a need in the Pakistani context.

## METHODS

This study is a secondary analysis from the baseline data from two studies^12^,^13^ aimed to measure the CTDs of nursing students in Islamabad-Pakistan. Data collection completed during May 2018-2020. Item generation and item reduction approach^14^ was employed to develop Pakistani-Critical Thinking Disposition Scale (PCTDS). In the item generation phase, constructs of the scale were identified through an in-depth literature review and items to measure the constructs were written by the first and second authors of this scale. Based on the assertions of Likert^15^ to measure the dispositions, a five-point agreement scale was incorporated (strongly agree=5, agree=4, somewhat agree=3, disagree=2 and 1=strongly disagree). Scores were set at underdeveloped (<50%), developing (50-79.99%), and well-developed (>80%) to discriminate critical thinking disposition levels.

The acquiescence bias was managed with 11-negatively worded items and those items were reverse coded. Committee method approach was employed to produce English-Urdu version of scale^16^ and was evaluated by multidisciplinary team. The items were evaluated on CVI requiring at least 0.40 coefficient. Validity, reliability and psychometric measures were employed through Lawshe guide^17^, using modified Kappa as good (0.60-0.74) and excellent (>0.74 ).^18^ Construct validity was measured based on classical test theory measures using maximum-likelihood exploratory factor analysis (MLEFA). Convergent validity was evaluated with average variance extracted and composite reliability value (>.30-.50) acceptable and (>.70) as good. Kaiser-Myer-Olkin (KMO) Bartlett’s tests was used for sample adequacy that requires value of >0.90 to proceed for exploratory and confirmatory factor analysis.^19^

Psychometric properties including root mean square of error of approximation (RMSEA) at, <0.08; parsimonious normed fit index (PNFI) and parsimonious comparative fit index (PCFI) at <0.5; Tucker-Lewis index (TLI), comparative fit index (CFI) and incremental fit index (IFI) at >0.9. The minimum coefficient of 3.0 for discrepancy function divided by degree of freedom (CMIN/DF) were evaluated. Internal consistency was evaluated with McDonald’s omega and Cronbach’s alpha was set at alpha and omega value of >0.70; the average inter-item correlation (AIC)^20^ 0.20-0.40 was determined as an acceptable measure of internal consistency. The error of scale score was calculated to determine the standard error of measurement with formula. The user-friendliness was evaluated by recruitment rate, successful completion rate, and time to complete the questionnaire. The analysis was performed with SPSS v21, Omega SPSS extension, and AMOS v21.

## RESULTS

The initial draft of PCTDS comprised of 54-items measuring seven constructs including contextual perspective-five-items, perseverance-seven-items, reflection-seven-items, intellectual integrity-nine-items, creativity-seven-items, open-mindedness-ten-items, and inquisitiveness nine-items. Items were generated in English and translated into Urdu language to make it user-friendly for Pakistani population. Bilingual version of the questionnaire was presented to field experts who were not part of the research team. They reviewed the questionnaire independently for language appropriateness, relevance of items with constructs, duplication, logical order, and need to omit or add items. Reviewers’ feedback was incorporated for language user friendliness and logical sequencing of items.

Few long items were shortened. Hence, 54-items for seven constructs were finalized with committee approach. All items received >.40 coefficient for CVI and modified Kappa, therefore they were retained. The KMO test value was 0.974 and Bartlett’s test (P-Value <0.001), therefore we proceeded to factor analysis. Based on factor loading indices i.e., item correlation coefficient <.40 and communality <0.20 six items were removed at this stage. The seven PCTDS constructs explained 73.37% of the total variance. While, contextual perspective 47.14% showed maximum and inquisitiveness 2.14% minimum respectively.

Initially, 47-items were evaluated in the first-order confirmatory factor analysis. The model fit indices calculated chi-square was 7105.18 (P-Value <0.001), CMIN/DF 0.102, and RMSEA 0.102 indicative of weak model fit at this stage. The parsimony adjusted measures were borderline while TLI, IFI, and CFI did not achieve the acceptable level. The five negatively worded and low correlated items affecting respective constructs were removed at this stage. The second-order CFA was performed on the 42-items which showed a better model fit chi-square 2035.61 (P-Value <0.001), CMIN/DF well under 3.0, RSMEA <0.08, parsimony indices >0.5, and baseline comparison indices >0.9 TLI, IFI, and CFI respectively.

The convergent validity indices, average variance extracted was 0.32, and composite reliability 0.95. The reliability indices revealed the internal consistency of the PCTDS with alpha and omega 0.869 and AIC 0.332. The standard error of measurement was 0.74. The scale also showed a high recruitment and completion rate of 98.8%. The average time taken to complete the 42-item questionnaire was 13±2 minutes.

**Table-I:** Factors and factor loading of the PCTDS (n=580).

Factor	Item	Factor Loading	h ^2^	Variance %
*Contextual Perspective*	I gather maximum information about a matter before making a final decision.	.540	.498	47.14
	The best way to solve a problem is to understand its complete background.	.413	.629	
	I analyze the pros and cons of every situation.	.539	.418	
*Perseverance*	Setbacks and hurdles cannot stop me from achieving my goal.	.481	.513	11.22
	I make every effort to overcome challenges.	.615	.491	
	*New projects shift my focus from previous ones.	.541	.372	
	Obstacles motivate me to succeed.	.552	.549	
	Once I begin something, I find ways to complete it.	.592	.534	
*Reflection*	I often review my ideas.	.489	.432	4.94
	I am able to deal with my doubts and uncertainties.	.590	.479	
	*I tend to make decisions very quickly.	.527	.601	
	I evaluate suggestions/opinions before making a decision.	.584	.578	
	I think carefully before I speak.	.460	.429	
*Intellectual Integrity*	I am consistent in my beliefs and actions.	.577	.548	3.06
	I avoid being biased at all costs.	.478	.531	
	Integrity is one of my core values.	.545	.494	
	Before judging a problem, I take into account the overall related information.	.668	.542	
	I make decisions based on reliable data.	.539	.503	
*Creativity*	I seek innovative ideas when confronted with a problem.	.455	.221	2.62
	I love to work on new or original ideas. I love to work on new or original ideas.	.987	.977	
	I instinctively deal with unexpected events.	.984	.970	
	I prefer to think differently from others.	.983	.966	
	I usually come up with an idea that doesn’t occur to others.	.979	.959	
*Open-Mindedness*	I embrace new ideas without resistance.	.978	.959	2.25
	*I diligently follow the practices of my seniors.	.974	.950	
	Suggestions from peers always add value to my work.	.979	.962	
	I accept change as a challenge.	.985	.974	
	I can easily see the world from other people’s perspectives.	.973	.947	
	*It is gratifying when others follow my point of view without question.	.969	.943	
	*I do not question the socio-cultural norms of my society.	.965	.934	
	*I feel extremely embarrassed if I have to admit my fault in front of others	.961	.932	
	I turn my mistake into an opportunity to learn.	.983	.970	
	I am open to constructive criticism.	.976	.957	
*Inquisitiveness*	I am always eager to learn more.	.988	.979	2.14
	I often learn through careful observations.	.988	.977	
	Questioning helps to improve my understanding.	.989	.981	
	I can listen to others patiently.	.982	.965	
	When something happens, I am curious about its process.	.985	.973	
	When I have a question, I always try to get the answer.	.986	.973	
	I enjoy trying to solve complicated problems.	.981	.969	
	I continually look for pieces of information related to solving a problem.	.987	.979	
	When I see the world, I see it with a questioning mind.	.982	.965	

h^2^ = communality,

* reverse coded items.

**Fig.1:**
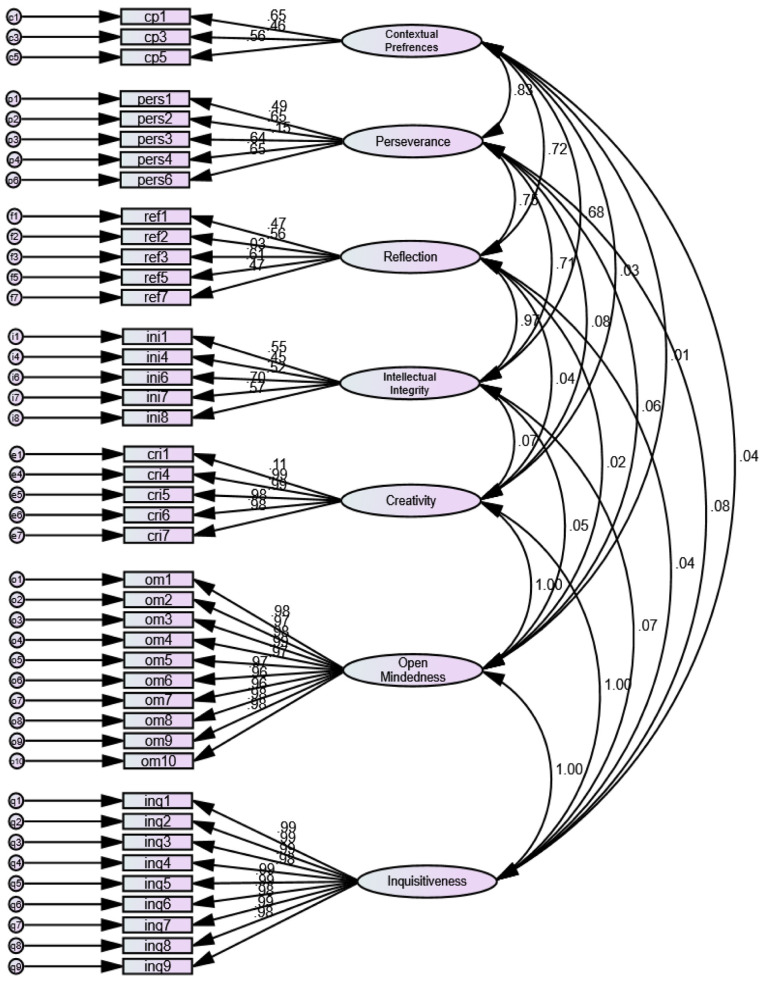
PCTDS constructs; modified model of second order confirmatory factor analysis.

**Table-II:** Model Fit indices of first and second-order confirmatory factor analysis.

Indices	X^2^	df	Sig.	CMIN/DF	RMSEA	PNFI	PCFI	TLI	IFI	CFI
1^st^ Order CFA	7105.18	1020	0.001	6.96	0.102	0.761	0.779	0.847	0.862	0.861
2^nd^ Order CFA	2035.61	798	0.001	2.55	0.052	0.843	0.858	0.968	0.971	0.971

## DISCUSSION

The final version of PCTDS comprised 42-items under seven constructs has shown favorable validity and reliability to measure CTDs among nursing students. The KMO and Bartlett’s test favorably justified the application of item-reduction by CFA in the current study. The findings are consistent with CTDS validation studies^7^,^21^ while other studies,^6^,^9^,^22^ did not report sample adequacy indices. Model fitness was achieved at the second CFA order following omission of one item from perseverance, two from reflection, and two from intellectual integrity. The model fit explained 73.37% of the total variance which is higher than another study of CTDS.^9^ This difference may be attributed a high variance (47.14%) in the construct of contextual perspective in our study. Since none of the previous CTD scales^5^-^9^ used contextual perspective, we retained this construct for improvement in future research.

In contrast with contextual perspective, the construct of inquisitiveness in our scale revealed least variance (2.14%) that is ground in perfect inter-item and inter-construct consistency. Therefore, retention of all items is justified in this construct. Unlike the PCTDS, a previous scale of CCTDI^5^ has two items in their construct of inquisitiveness. Although inquisitiveness, open-mindedness, and creativity showed perfect AIC, weak to moderate AIC was noted with intellectual integrity, reflection, perseverance, and contextual perspective in the first and second-order CFA. Further, the standard error of measurement was high which may due to disparity between the constructs with regard to AIC, and similarly worded items.

The standard error of measurement was not reported in international studies validating CTD scales.^5^-^7^,^9^,^21^,^22^ Use of this measure improved the scale on second-order CFA. The reliability indices including Cronbach’s alpha, McDonald’s omega, and AIC showed highly favorable internal consistency (.879). This finding is comparable to extensively used CCTDI^5^ reporting 0.70-.90 alpha value in different studies. Hence, PCTDS is equally reliable scale to measure CTDs. Furthermore, alpha values of PCTDS is higher than CTDS,^6^ YCTDS,^9^ and RCTDS.^21^

### Limitations

The tool was tested only among undergraduate nursing students. The contextual perspective construct may be revised in future research to reduce the variance.

## CONCLUSION

PCTDS is a valid and reliable scale to measure the critical thinking dispositions of nursing students. Measuring CTDs is a stepping-stone to develop CT skills. Critical thinking encompassing CTDs is an emerging competence for nursing students as well as for other healthcare professionals. Therefore, researchers foresee the testing and use of this scale in nursing and other healthcare disciplines.

### Authors’ Contribution:

**RG:** Conception, item writing, intellectual revisions, accountable for accuracy and integrity of work.

**NS:** Conception, item writing, and acquisition of data,

**GV:** Analysis, interpretation of findings and drafting of manuscript.

**AG:** Critical revision and data interpretation.

**HR:** Acquisition of data.

All authors approved final version for publication.
